# Genetic structure of the white-footed mouse in the context of the emergence of Lyme disease in southern Québec

**DOI:** 10.1002/ece3.620

**Published:** 2013-06-03

**Authors:** Anita Rogic, Nathalie Tessier, Pierre Legendre, François-Joseph Lapointe, Virginie Millien

**Affiliations:** 1 Redpath Museum, McGill University859 Sherbrooke Street W., Montréal, Québec, H3A 0C4, Canada; 2 Département de sciences biologiques, Université de MontréalC.P. 6128, Succ. Centre-ville, Montréal, Québec, H3C 3J7, Canada

**Keywords:** Barriers, climate change, fragmentation, gene flow, Lyme disease, *Peromyscus leucopus*

## Abstract

The white-footed mouse (*Peromyscus leucopus*) has expanded its northern limit into southern Québec over the last few decades. *P. leucopus* is a great disperser and colonizer and is of particular interest because it is considered a primary reservoir for the spirochete bacterium that causes Lyme disease. There is no current information on the gene flow between mouse populations on the mountains and forest fragments found scattered throughout the Montérégie region in southern Québec, and whether various landscape barriers have an effect on their dispersal. We conducted a population genetics analysis on eleven *P. leucopus* populations using eleven microsatellite markers and showed that isolation by distance was weak, yet barriers were effective. The agricultural matrix had the least effect on gene flow, whereas highways and main rivers were effective barriers. The abundance of ticks collected from mice varied within the study area. Both ticks and mice were screened for the presence of the spirochete bacterium *Borrelia burgdorferi*, and we predicted areas of greater risk for Lyme disease. Merging our results with ongoing Lyme disease surveillance programs will help determine the future threat of this disease in Québec, and will contribute toward disease prevention and management strategies throughout fragmented landscapes in southern Canada.

## Introduction

The spirochete bacterium responsible for Lyme disease in humans was discovered in the 1970's in Lyme, Connecticut (U.S.A.). *Borrelia burgdorferi* is transmitted through the bite of the black-legged tick (*Ixodes scapularis*), an acarian from the Ixodidae family found throughout the eastern and central United States, and along the eastern margins of Mexico and Canada (Almazan-Garcia 2005). Its life cycle includes two immature stages (larval and nymph) followed by a reproductive adult stage. A single blood meal is required for transition into the next stage or for reproductive purposes for adult females. Although the ticks may feed on a wide range of vertebrate hosts, there are four species of small mammals (eastern chipmunk, short-tailed shrew, masked shrew, and white-footed mouse) that have a high reservoir competence for *B. burgdorferi* (Ostfeld 2011). Reservoir competence is determined by how susceptible the host is to the infection once bitten, how well the pathogen replicates inside the host, and how efficiently the bacterium is transmitted back to the vectors that are feeding on the host (LoGiudice et al. 2003). The white-footed mouse (*Peromyscus leucopus*) is considered to have the highest reservoir competence, infecting 75–95% of the larval ticks that feed on them (Mather et al. 1989; Ostfeld 2011). Once infected, the mice are asymptomatic and the infection has little impact on their activities (Schwanz et al. 2011).

Environmental changes are important drivers for the emergence of vector-borne diseases, including Lyme disease (Halos et al. 2010), and both forest fragmentation and global climate change have been shown to play a critical role in the spatial and temporal distribution of this disease (Ogden et al. 2008; Halos et al. 2010). Climate change allows reservoir hosts and vectors to expand their range into new territories (Brownstein et al. 2005). In addition, fragmentation of forested landscapes due to human activities affects local biodiversity by favoring habitat generalists and species that experience high population densities and small home ranges (Allan et al. 2003). A species matching this description is the white-footed mouse; an important reservoir for *B. burgdorferi* and host for *I. scapularis* (Eisen et al. 2012).

**Figure 1:**
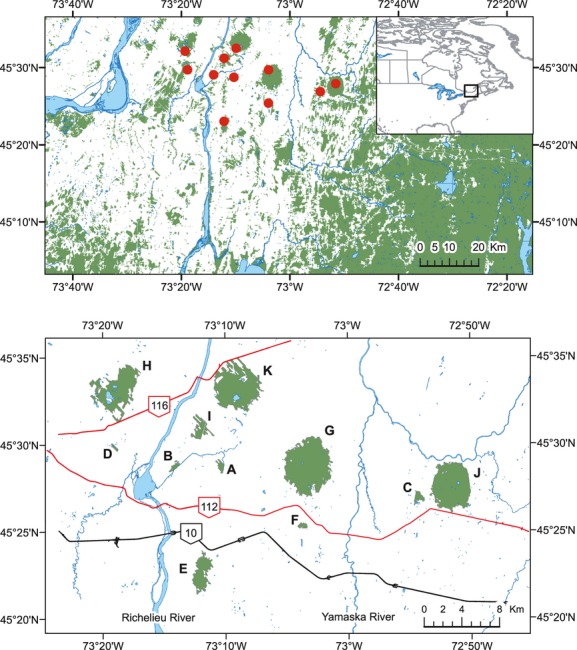
Geographic locations of the 11 study sites and the main geographic barriers among them. Four Monteregian hills were considered: Mont Saint Bruno (H), Mont Saint Hilaire (K), Mont Rougemont (G), and Mont Yamaska (J). In the top panel, a greater forest density is observed in the southern portion of the Montérégie (mapped with SIEF data, MRNQ 2000). Agricultural fields are shaded in white, forested habitats in light gray, and water bodies in dark gray.

Current global warming is expected to influence the emergence of Lyme disease in eastern Canada (Brownstein et al. 2005; Ogden et al. 2006, 2008; Leighton et al. 2012) and the Public Health Agency of Canada has already identified multiple locations in southern Canada, including Québec, as endemic for Lyme disease (Government of Canada, Public Health Agency of Canada 2012). Québec's southern region, the Montérégie (Fig. 1), is of particular interest because of its highly fragmented landscape and recent temperature increases. Over the last 40 years, the Montérégie has had a 0.8°C increase in its mean growing season temperature (Almaraz et al. 2008). Intense agricultural practices, which began in the 1940's, has led to a 70% decrease of the local forests (Wampach 1988) creating a landscape comprising the forested Monteregian hills that extend from Montréal to the United States border, and other smaller forest patches surrounded by an urban or agricultural matrix. Climate warming combined with the fragmentation of the landscape may have favored the white-footed mouse to expand its range further north into southern Québec over the last 15 years (V. Millien, pers. comm.).

White-footed mice are considered extreme generalists in terms of their dietary and habitat preferences (Ostfeld and Keesing 2000). Although forest fragmentation generally reduces species diversity within remnant patches (Allan et al. 2003), numerous studies found that *P. leucopus* population densities increased as patch size decreased, indicating that their abundance is actually enhanced by forest fragmentation (Yahner 1992; Nupp and Swihart 1996, 1998; Krohne and Hoch 1999; Mossman and Waser 2001; Wilder et al. 2005; Anderson and Meikle 2010). The reason for this inverse relationship is not yet entirely understood. One notion is that patch edges may support greater vegetation density and complexity, thus resulting in greater food availability. As smaller forest patches have a greater perimeter-to-area ratio than larger patches, mouse densities will grow with the increased edge habitat (Wilder et al. 2005; Klein and Cameron 2012). In addition, species diversity decreases with patch size. The mice thus experience less interspecific competition and predation pressure in smaller forest patches (Ostfeld and Keesing 2000). Finally, small forest patches are usually surrounded by areas of less suitable habitats, which may inhibit emigration. If mice residing within these “patch islands” have a difficult time dispersing to new environments, their populations will soar within the small patches (Nupp and Swihart 1998; Klein and Cameron 2012).

Studies showed that *P. leucopus* does not always conform to the source–sink model either (Morris and Diffendorfer 2004; Grear and Burns 2007; Linzey et al. 2012). The white-footed mouse is a woodland species, but is also known to thrive in small forest fragments (Barko et al. 2003), therefore we tested for a source–sink relationship between mountains (source) and their closest forest fragment (sink) to observe whether the mice in our system conform to the model. Although the source–sink model predicts the direction of dispersal, there have been conflicting reports on how difficult dispersal may be when traveling between isolated forest habitats (Anderson and Meikle 2010).

*Peromyscus leucopus* is a great disperser and colonizer, and a single mating pair may establish a successful population upon entering a new habitat (Cummings and Vessey 1994). They have been found to expand their range at an astonishing rate: approximately 15 km/year in the Great Lakes region (Myers et al. 2005, 2009) and 10 km/year in southern Québec (V. Millien, pers. comm.) but their local movements may get hindered by various landscape barriers including agricultural fields (Krohne and Hoch 1999; Klein and Cameron 2012), water bodies (Savidge 1973; Loxterman et al. 1998; Klee et al. 2004), and roads (Merriam et al. 1989; Clark et al. 2001; McGregor et al. 2003; McLaren et al. 2011). Other studies indicate, however, that *P. leucopus* shows high levels of dispersal regardless of their surroundings (Middleton and Merriam 1981; Wegner and Merriam 1990; Mossman and Waser 2001). If the dispersal of *P. leucopus* populations in Montérégie was unhindered by environmental barriers, Lyme disease would have the potential to spread fairly quickly throughout southern Québec.

Because of the complexity of their transmission mechanisms, population genetic studies have become an important tool to help understand the spread of zoonotic diseases and develop effective control strategies (Wilcox and Gubler 2005). Limited migration would allow for more detailed management strategies (e.g., reduction of specific populations), while wide dispersal would indicate the need for large-scale treatments (Tabachnick and Black 1995). Since 2003, Québec's health practitioners have been obligated to report any Lyme disease diagnoses. Since then, multiple indigenous-born cases of Lyme disease have been confirmed and approximately 10% of the ticks found in Québec are infected with *B. burgdorferi* (Trudel and Serhir 2009). Studies also showed that of all the Canadian regions investigated, Québec had the most newly established tick populations (Leighton et al. 2012). It is predicted that *I. scapularis* numbers in southern Québec will double by 2020 due to climate warming (Ogden et al. 2006). The threat of a northern range expansion of *I. scapularis* coupled with the current northern expansion of *P. leucopus* in Montérégie significantly increases the probability of humans becoming infected by *B. burgdorferi*. White-footed mice play an important role in the spread of the bacteria responsible for Lyme disease, yet there is no current information on (i) whether the mice are establishing successful populations on the mountains and forest fragments found scattered throughout Montérégie, (ii) the gene flow between these habitable sites, and (iii) the relative effects of various landscape barriers on mouse dispersal.

In this study, we identified the processes that account for the current level and distribution of the genetic variation in populations of *P. leucopus* in southern Québec. With the enhanced dispersal abilities and generalist behavior of the white-footed mouse, we hypothesized that its movements across the fragmented landscape of Montérégie would not be structured in space and would not be affected by geographic distance or barriers. We conducted a population genetics study of 11 *P. leucopus* populations and incorporated *I. scapularis* data collected from each mouse to evaluate the strength of the relationships between *P. leucopus* gene flow, the black-legged ticks, and the threat of Lyme disease in the region. Our results are pertinent not only to help determine the future threat of Lyme disease in Québec, but also to contribute toward disease prevention and management strategies throughout the province and the rest of southern Canada.

## Materials and Methods

### Study sites

We trapped white-footed mice from 11 different sites in the Montérégie region, southern Québec (Fig. 1, Table 1). Four sites (G, H, J, and K) are part of the Monteregian Hills. The other sites are forest fragments located between those hills. Overall, the study area covers 634 km^2^. Individual site areas ranged from 0.14 to 21.4 km^2^ (Table 1). Four of the fragments (C, D, F, and I) were selected for their proximity to the mountains (less than 3 km). The other three forest fragments (A, B, and E) formed a connection from the south to the north of the study area.

**Table 1:** Location, elevation, and sample size for the study sites

Site	Latitude	Longitude	Elevation (m)	Nearest mountain	number *Peromyscus leucopus*	Years collected
A	N45 28.91	W73 10.47	44	NA	35	2010, 2011
B	N45 29.01	W73 13.86	23	NA	32	2010, 2011
C	N45 26.97	W72 54.54	83	Mont Yamaska	34	2011
D	N45 29.75	W73 18.99	35	Mont Saint Bruno	31	2011
E	N45 23.28	W73 12.09	63	NA	34	2010, 2011
F	N45 25.43	W73 03.92	68	Mont Rougemont	35	2011
G	N45 29.74	W73 04.06	89	NA	35	2009, 2011
H	N45 32.97	W73 18.23	90	NA	37	2009
I	N45 31.23	W73 12.14	59	Mont Saint Hilaire	35	2011
J	N45 27.42	W72 51.52	295	NA	21	2009, 2011
K	N45 33.16	W73 09.09	244	NA	38	2007–2009

Nearest mountains for fragments are indicated. G, Mont Rougemont; H, Mont Saint Bruno; J, Mont Yamaska; K, Mont Saint Hilaire.

### Field sampling

Sherman live traps were used for small mammal captures and a total of 367 white-footed mice were collected, with numbers ranging from 30 to 38 at each site, except for Mont Yamaska where only 21 mice were trapped (Table 1). All specimens were deposited in the McGill's Redpath Museum collection (Montréal, Québec, Canada). Each mouse was searched for the presence of ticks which were then removed, counted, and assigned to larval, nymph, or adult life stages. All mice and ticks were then screened for the presence of *B. burgdorferi* following the method of Ogden et al. (2011).

### DNA extraction and species identification

DNA was extracted from the liver tissue of the mice trapped in 2007–2009 using the Invitrogen™ Purelink™ Genomic DNA Kit (Version A, February 2007) (Invitrogen™ Inc., Burlington, Canada). The following changes were made to the Mammalian Tissue and Mouse/Rat Tail Lysate protocol: we lysed the tissue overnight at 37°C and increased the centrifuge time to 2 min for the elution step. Liver tissue from the mice collected from 2010 to 2011 was extracted using a standard 3-day phenol/chloroform extraction procedure as described by Sambrook et al. (1989). Because there are two coexisting species of *Peromyscus* in our study area (*P. leucopus* and *Peromyscus maniculatus*), all the mice were identified to species using species-specific primers from the mitochondrial COIII sequence, as described in Tessier et al. (2004). Only *P. leucopus* were considered for further analyses.

### Microsatellite amplification

Microsatellite markers selected were: *PMl*01, *PMl*03, *PMl*04, *PMl*05, *PMl*06, *PMl*09, *PMl*11, and *PMl*12 (Chirhart et al. 2005) as well as *PLGT58, PLGT66,* and *PLGATA70* (Schmidt 1999). Polymerase chain reactions differed between primer sets (Appendix S1). Amplification success was determined by staining the PCR products with SYBR® Green I nucleic acid gel stain (Invitrogen™ Inc., Burlington, Canada), running them on a 2% agarose gel, and revealing the bands under UV light. Fourteen percent of all individuals were independently amplified and genotyped twice to confirm the observed genotypes. All PCR products were sent to the McGill University Génome Québec Innovation Centre for genotyping using an ABI-3730XL DNA Analyzer, and all alleles were scored visually three separate times using GeneMarker v. 1.91 (SoftGenetics® LLC, PA).

### Genetic variation

Within-population genetic diversity was estimated by testing for linkage disequilibrium, for departures from Hardy–Weinberg equilibrium, and by computing expected (*H*_*E*_) and observed heterozygosity (*H*_*O*_) using GENEPOP 4.0 (Raymond and Rousset 1995; Rousset 2008). Allelic richness (*A*), the number of private alleles (*p*), and the mean number of alleles per locus within each population (*k*) were computed using FSTAT 2.9.3 (Goudet 1995). MICROCHECKER 2.2.3 (Van Oosterhout et al. 2004) helped detect scoring errors and genotyping artifacts in the data. If null alleles were detected, we estimated their frequency following the method of Kalinowski and Taper (2006).

### Population genetic structure

We performed Fisher's exact tests on the 11 loci under study using GENEPOP 4.0 to determine the significant differences in allele frequencies between populations. Genetic differentiation of populations was also evaluated by calculating pairwise *F*_ST_ values using Arlequin 3.5 (Excoffier et al. 2005); significance levels were assessed through a permutation test with 1000 iterations.

We used STRUCTURE 2.3 (Pritchard et al. 2000) to determine the most likely number of genetically distinct clusters (*K*) of populations using a length of burn-in period at 100,000 and the number of Markov Chain Monte Carlo replicates after burn-in at 1100,000. Posterior probabilities were calculated for *K* = 1 to *K* = *n* + 1, where *n* = number of populations sampled, using the *no admixture model*, which is better at detecting subtle structures (Pritchard et al. 2010). Allele frequencies were assumed independent among populations and calculations were repeated 10 times for each value of *K*. The optimal number of populations to which the individuals were assigned was the value of K associated with the highest ΔK value (Evanno et al. 2005; Martien et al. 2007). We also performed hierarchical agglomerative clustering based upon the *F*_ST_ indices of differentiation, using the agnes function of the “cluster” package (Maechler 2012) in R (R Development Core Team 2012) to construct a unweighted pair group method with arithmetic mean (UPGMA) tree and compute its agglomerative coefficient. This coefficient, ranging from 0 to 1, describes the strength of the clustering structure with a value close to 1 indicating a strong structure (Kaufman and Rousseeuw 1990).

To assess the effect of geographical distances on the genetic structure of *P. leucopus* populations, we carried out an isolation-by-distance analysis using IBDWS 2.3 with 10,000 permutations (Jensen et al. 2005). We then carried out a canonical redundancy analysis (RDA) using the rda function of the “vegan” package (Oksanen et al. 2012) in R (R Development Core Team 2012) to determine whether the two rivers (the Richelieu and Yamaska Rivers) and three major highways (Highways 10, 112, and 116) in the study area acted as dispersal barriers for the mice. An RDA was preferred over Mantel tests because it has been shown to have much greater statistical power (Legendre and Fortin 2010). A series of dummy variables [0, 1] were used to characterize whether sites were on one side or the other of each barrier. *F*_ST_ values were used to compute a principal coordinate analysis (PCoA). Several eigenvectors corresponded to negative eigenvalues, therefore we computed a second PCoA by using the square root of the genetic distances. Any remaining eigenvectors corresponding to negative eigenvalues were omitted from further analyses as they represented less than 5% of the total variance. The PCoA eigenvectors were then used as the matrix of response variables in RDA (distance-based RDA approach, Legendre and Anderson 1999). The model was tested for significance using 1000 random permutations. A second RDA was carried out using the allele frequencies of each population as the matrix of dependent variables to ensure that the same barrier effects were observed for both datasets, and significance was tested using 1000 random permutations as well. Models with the lowest Akaike Information Criterion (AIC) were selected; AIC are measures of the goodness of fit of the data to an estimated statistical model (Legendre and Legendre 2012). To further evaluate how agriculture affected dispersal, we performed Fisher's exact tests and calculated pairwise *F*_ST_ values on the five populations not separated by highways or water bodies (Sites A, B, I, K, and G, Fig. 1).

We calculated the effective number of migrants (*Nm*) between mountains and their closest forest fragment patch using MIGRATE 3.2.19 (Beerli 2002) to test whether the different populations adhere to the source–sink hypothesis. We used a single stepwise mutation model and assumed a migration matrix model with variable theta (Θ) and a constant mutation rate for all loci. Start values for the migration parameter were generated from the *F*_ST_ calculations. To ensure that the gene flow estimates were accurate, MIGRATE was run three times to verify that final chains were estimating similar values for Θ and for 4*Nm*. Values of 4*Nm* were divided by four to compare levels of gene flow between populations (i.e., *Nm*), which were also summed to provide estimates for the overall immigration and emigration rates of each population.

We tested the source–sink model further using the ratio of private alleles between sites to calculate the gene flow symmetry (GFS) index, as described by Fedorka et al. (2012). GFS values were calculated using the PopGenKit package (Rioux Paquette 2011) in R (R Development Core Team 2012). Standard errors were estimated with 1000 bootstrap replicates (Efron and Tibshirani 1993). Symmetrical gene flow would yield a GFS index of 1, whereas asymmetrical gene flow from site *i* to *j* would yield a GFS index <1 or >1 if asymmetrical from site *j* to *i*. Finally, we conducted an assignment test using GENECLASS2 (Piry et al. 2004) where the assignment of individuals to populations was carried out using the Bayesian algorithm of Rannala and Mountain (1997). We considered the 11 populations as independent units to help determine recent estimates of migration. In all tests where multiple comparisons were made, probability values were adjusted with sequential Bonferroni corrections (Rice 1989).

## Results

### Ticks

Approximately half of the mice trapped at sites C, D, and E were carrying ticks (Table 2). They also hosted the largest number of ticks per mouse compared to all other sites. Site E was the southernmost population in our study and sites C and D were located at the extreme east and west ends of the study area, respectively. All other sites had considerably lower numbers of mice carrying ticks as well as number of ticks per mouse.

**Table 2:** Tick abundance collected from mice

Site	Number of mice	Mice with ticks		Minimum and maximum number of ticks		
	
		#	%	Larvae	Nymphs	Adults
A	35	1	2.9	0	1	0
B	32	2	6.3	1	0	0
C	34	14	41.2	1–7	1	0
D	31	16	51.6	1–8	0	0
E	34	21	61.8	1–7	1	0
F	35	3	8.6	2	1	0
G	35	0	0	0	0	0
I	35	3	8.6	1–3	0	0
J	21	2	9.5	1–2	0	0
Total	292	62	21.2			

Sites H and K were not sampled for ticks. Three infected mice and an infected tick were collected from site D in 2011.

### Lyme disease

Only three of the 367 trapped mice were positive for *B. burgdorferi*. These mice were all collected at site D. Of the 149 total ticks collected off the mice, one tick was found to be positive for the bacterium. It was originally removed from one of the infected mice from site D.

### Mice – genetic variation

Over the entire data set, seven locus pairs were in linkage disequilibrium out of 605 pairs. These significant values occurred in five of the 11 populations but with no apparent pattern (i.e., no locus pairs were consistently in linkage disequilibrium). All loci were thus considered independent in further analyses. Hardy–Weinberg equilibrium (HWE) tests detected five deviations out of 121 tests occurring in four different populations at four different loci (Site A = *Pml*01; Site F = *Pml*03 & *Pml*06; Site H = *PMl*09; Site J = *PMl*03). Although assortative mating or high levels of genetic drift may have induced these deviations from HWE, we also considered the possibility of the presence of null alleles in the data. MICROCHECKER 2.2.3 revealed that *Pml*09 may contain null alleles at site H while *Pml*03 was likely to contain null alleles at sites E, F, G, and J. The estimated null allele frequencies for the *Pml*09 locus were low (0.079–0.098), and as it was considered to possibly have null alleles at site H only, we kept this marker in the analysis. *Pml*03, on the other hand, was considered likely to contain null alleles in four of the 11 populations, and estimated null allele frequencies ranged from 0.08 to 0.22. Sites whose *Pml*03 locus departed from HWE (F and J) ranged on the higher end of the estimated null allele frequency spectrum (0.21 and 0.22, respectively). We thus conducted both intra- and interpopulation analyses with and without the *Pml*03 locus and found no statistical differences between the results of the two analyses. We therefore retained the *Pml*03 marker in our study.

The 11 loci were polymorphic and all populations were highly variable (Table 3), with the number of alleles per microsatellite ranging from 14 to 29. This high number of alleles per marker is consistent with other *P. leucopus* population genetic studies (Mossman and Waser 2001; Chirhart et al. 2005; Anderson and Meikle 2010; Munshi-South and Kharchenko 2010; Munshi-South 2012).

**Table 3:** Sample size (*n*), mean number of alleles per locus (*k*), allelic richness (*A*), number of private alleles (*p*), and observed (*H*_*O*_) and expected heterozygosity (*H*_*E*_) for the 11 populations

Site	n	*k*	*A*	*p*	*H*_*O*_	*H*_*E*_
A	35	13.7	10.7	4	0.858	0.860
B	32	11.3	9.4	4	0.846	0.844
C	34	13.3	10.7	13	0.853	0.852
D	31	11.8	9.7	2	0.902	0.830
E	34	12.0	10.0	3	0.917	0.863
F	35	13.2	10.7	9	0.874	0.869
G	35	11.9	10.2	2	0.893	0.868
H	37	10.6	9.1	3	0.861	0.853
I	35	12.1	9.9	3	0.891	0.846
J	21	9.8	9.4	0	0.872	0.845
K	38	11.8	9.7	4	0.832	0.842
Mean					0.873	0.852

Of the 241 alleles, 47 were private alleles found at low frequencies (Table 3). The highest number of private alleles (*p*) were found at sites C and F (13 and 9 alleles, respectively). All other sites had 0 to 4 private alleles. Allelic richness (*A*) was similar across all populations and ranged from 9.1 to 10.7.

The mean observed and expected heterozygosity values (*H*_*O*_ and *H*_*E*_) were estimated at 0.873 (0.832–0.917) and 0.852 (0.830–0.869), respectively (Table 3). The high heterozygosity values across all populations revealed high polymorphism among the 11 populations.

### Mice – population genetic structure

Fisher's exact tests indicated that the two populations located to the west of the Richelieu River (sites D and H) exhibited the highest degree of allele frequency differentiation (Table S1). Sites D and H had 49 and 48 significant differences out of 110 pairwise comparison tests, respectively, yet allele frequencies were similar between them (1/11 significant difference). Besides Mont Saint Bruno (site H) to the west of the Richelieu River, the other three mountains included in this study had low allelic differentiation when compared with other sites. Mont Rougemont (site G) had 11 significant differences, Mont Yamaska (site J) had 12 significant differences, and Mont Saint Hilaire (site K) had 21 significant differences out of 110 pairwise comparison tests performed for each population. Allelic differentiation in the remaining forest fragments ranged from 18 to 27 significant differences for each population.

*F*_ST_ values ranged from 0 to 0.054, and the majority of the values were significant (41 out of 55) (Table S1). Site D and H were the most distinct populations exhibiting differences from all other populations. Site C also exhibited differences from all other populations except for site J. Site E differed from all other populations except for site F which differed from all populations except for sites E and G. The remaining populations did not exhibit any clear pattern. For the five populations separated by agricultural fields only (sites A, B, G, I, and K), only a few *F*_ST_ values were significant (2 out of 10 values) and allelic differentiation was very low (from 0 to 3 significant differences out of 44 pairwise comparison tests), indicating high levels of gene flow among these sites.

**Figure 2:**
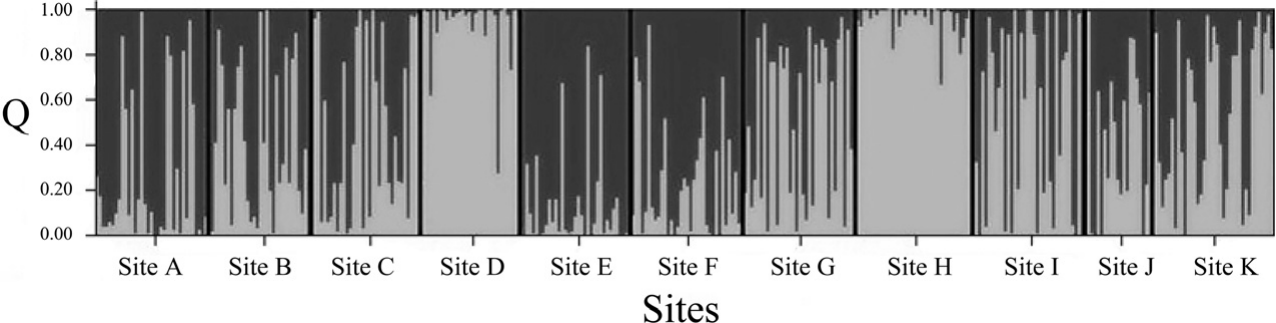
STRUCTURE's population clustering analysis suggesting two population clusters (*K* = 2). Each line represents an individual, and Q is the proportion of the individual's genome which is part of each *K* subpopulation.

**Figure 3:**
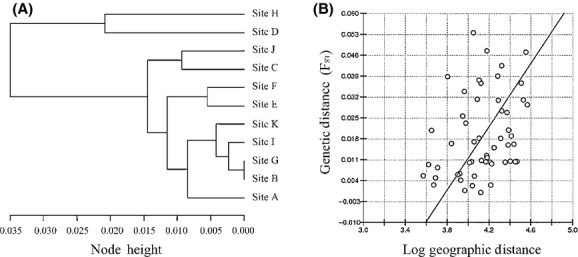
Results of the (A) hierarchical clustering tree (UPGMA) and (B) the isolation-by-distance analysis using *F*_ST_ values.

We found two distinct population clusters using STRUCTURE (Fig. 2 and S1, Table S2). One cluster corresponded to the sites located to the west of the Richelieu River (sites D and H), and the second cluster corresponded to all other sites. Within the second cluster, sites E and F showed some differentiation from all other sites, but were not well defined. The UPGMA tree showed a similar separation of sites D and H from all other populations as well as the separate clustering of sites E and F (Fig. 3A). Sites C and J (located to the east of the Yamaska River) also clustered together, and any remaining sites grouped together into a single cluster (agglomerative coefficient = 0.78).

We found a significant pattern of isolation by distance among the 11 populations. The pairwise genetic distances (*F*_ST_) increased with geographic distance (slope = 0.053, *r*_Mantel_ = 0.32; *P* < 0.04) (Fig. 3B).

### Barrier effect

Forward selection in RDA analysis using either the principle coordinates derived from the *F*_ST_ values or the allele frequencies as the dependant variables, and geographic barriers as the explanatory variables, revealed four significant barriers in the study area which in order of importance were: the Richelieu River, the Yamaska River, and highways 116 and 112 (Table 4). Although interpopulation genetic structure analyses did not show the Yamaska River and highways 116 and 112 to isolate mouse populations as effectively as the Richelieu River, the RDA analyses indicated that these barriers are effective at slowing dispersal. The largest river (Richelieu) was the strongest determinant of genetic differentiation, followed by the smallest river (Yamaska), and finally by the two highways (116 and 112).

**Table 4:** Canonical redundancy analyses with *F*_ST_ values and allele frequencies as the dependant variable and dispersal barriers as independent variables

	AIC	F	Adjusted *r*^2^
*F*_ST_			
Richelieu River	−53.8	6.41*	
Yamaska River	−54.8	2.50*	
Highway 116	−55.8	2.19*	
Highway 112	−58.4	3.17*	0.63
Allele frequencies			
Richelieu River	−12.3	3.18**	
Yamaska River	−12.3	1.60*	
Highway 116	−12.4	1.54**	
Highway 112	−13.0	1.59*	0.33

AIC, *F*, and adjusted *r*^2^ values are shown for each model.

^ns^*P >* 0.05,

* *P* < 0.05,

** *P* < 0.01.

### Source–sink and migration

Using MIGRATE, we found low *Nm* values (0.18–0.43) between populations and low overall immigration and emigration rates (Table S3). Emigration rates from mountain populations into their closest fragments were similar to the emigration rates from fragment populations into mountains. Immigration rates also did not show any apparent patterns supporting the source–sink hypothesis. Lastly, there was no significant relation between pairwise migration rates and geographic distance between sites (*P* = 0.2). However, we detected asymmetrical gene flow for two mountain-fragment pairs (from site D to H and from site C to J) with a GFS index greater than one (Table S4).

The success rate of assigning individuals to their population of origin ranged from 59% to 97% (Table S5). Sites B and I were the only populations whose assignment success rate dropped below 70%, with 19% and 20% of their individuals (respectively) assigned to site G.

## Discussion

As a widespread, generalist and opportunistic species, we hypothesized that the white-footed mouse would not display any strong spatial genetic structure. Surprisingly, we found that the mice in Montérégie were, to some degree, structured in space. The populations exhibited high heterozygosity levels, which is not uncommon for *Peromyscus* mice found within fragmented or isolated habitats (Mossman and Waser 2001; Chirhart et al. 2005; Munshi-South and Kharchenko 2010). Although isolation by distance was significant for the 11 populations, this relation was weak. The remaining variability in the data was largely due to four of the five barriers present between populations in the study area: the Richelieu River, the Yamaska River, and highways 116 and 112.

To a certain degree, white-footed mice are able to swim (Klee et al. 2004), yet water bodies only 3–4 m wide (Savidge 1973), rivers (Klee et al. 2004) and sea armlets between islands (Loxterman et al. 1998) have been found to be significant dispersal barriers. Here, the Richelieu River was the strongest barrier isolating the two sites to the west of it. These sites showed consistent and strong genetic differentiation from all other sites in all analyses.

The second main water body in our study area, the Yamaska River, was also a significant dispersal barrier but had less influence on dispersal compared to the Richelieu River. When considering the Montérégie region on a larger scale, its southeastern landscape is composed of a greater number of forest fragments than what is observed in our study area (Fig. 1), thus possibly creating an effective southern connection for dispersing mice. White-footed mice may cover a very wide range of dispersal distances, from 85 to 867 m, throughout their lifespan (Krohne et al. 1984), with some extreme dispersers found 5–14 km away from their original site of capture (Maier 2002). Large continuous tracts of forest significantly increase white-footed mouse dispersal in comparison to fragmented forests (Krohne and Hoch 1999). Therefore in our study area, mice may have the potential to disperse over large distances around the Yamaska River by using the large number of forest fragments found in the southern part of the Montérégie region.

Mont Yamaska was also the only study site where 13 of the 34 mice we trapped were *P. maniculatus*, the deer mouse. The white-footed mouse and deer mouse are both highly abundant throughout North America (Dewey and Dawson 2001), however, the white-footed mouse has expanded its northern range limit into northern USA (Myers et al. 2005, 2009) and southern Québec over the last few decades. Although abundant in the past (Grant 1976), the deer mouse seems to have drastically declined in abundance to the point that most of the *Peromyscus* mice we caught in Montérégie were *P. leucopus*. Montérégie is part of the Great Lakes and St. Lawrence River forest system (D'Orangeville et al. 2008), which is considered as a transitional zone between the deciduous forests of eastern North America and the boreal forest in northern Canada (Ontario Ministry of Natural Resources 2012). Although both species of mice are found in a variety of habitat types, *P. maniculatus* favors boreal forests (Garman et al. 1994; Myers et al. 2005, 2009) and shows better adaptation to cold weather than *P. leucopus* (Pierce and Vogt 1993). This adaptation may explain the relative larger abundance of the deer mouse on Mont Yamaska, which had a greater number of boreal tree species than all other sites, and was the tallest of the four mountains included in this study. Recent climate warming in the Montérégie region may have pushed the deer mice further north, yet a population may have remained on Mont Yamaska where cooler temperatures and a boreal microhabitat provided a suitable habitat for this species. As boreal habitats are less optimal for *P. leucopus,* these sites are important to take into consideration when analyzing the dispersal of *P. leucopus* throughout southern Québec.

The highways in our study system slowed mouse dispersal but were not absolute barriers. White-footed mice have been found to use a large array of corridor types when moving between fragmented forest habitats including roadside ditches (Cummings and Vessey 1994), fences lined with vegetation (Merriam and Lanoue 1990), and stone walls (Parren and Capen 1985). Merriam and Lanoue (1990) found that the mice used all categories of fencerows as corridors, from simple fencerows less than 1 m in width with less than 10% of vegetative cover, to complex fencerows wider than 2 m with trees and full ground cover present in more than 10% of the fencerow's length. This flexibility in types of dispersal corridors used may present highways as less imposing barriers for the mice to cross compared to large rivers.

Agricultural fields had the least effect on white-footed mouse dispersal in our study area. Previously published results are conflicting: agricultural fields have been found to act as significant dispersal barriers (Krohne and Hoch 1999; Klein and Cameron 2012), as seasonal barriers where crop height and maturity determine whether they are used as dispersal routes (Cummings and Vessey 1994; Anderson et al. 2006), or not as barriers at all (Middleton and Merriam 1981; Mossman and Waser 2001). Since we trapped mice between the months of July and September when crops were high enough to provide adequate cover and food for migrating mice, it is possible that the fields were used as seasonal corridors between forest patches. Whether they are used seasonally or throughout the year, the high levels of gene flow observed in our study suggest that the agricultural matrix may allow the mice to disperse effectively between sites. These results support our original hypothesis that mouse populations lacking major roads or water bodies between them would not be genetically structured.

The mouse populations in our study system did not conform to the source–sink model, which is not uncommon for white-footed mice (Morris and Diffendorfer 2004; Grear and Burns 2007; Linzey et al. 2012), and sink patches have been found to regulate themselves if individuals are interactive and resident (Linzey et al. 2012). Gene flow asymmetry was nevertheless detected between certain mountain-to-fragment populations when considering the ratio of private alleles. This may indicate subtle gene flow from source to sink populations, but *Nm* values were not high enough to assume recurrent extinction and recolonization of sink populations by their closest source.

Our study was designed to better understand the pattern of the spread of Lyme disease within the Montérégie region, and to help identify areas of greater risk for human populations. Different types of landscape barriers varied in their effectiveness as disrupters to *P. leucopus* dispersal. Agricultural fields may pose the greatest challenge to Lyme disease management strategies, as they did not hinder gene flow between sites as effectively as the other barriers. Québec's southwestern landscape has largely been developed for farming purposes, resulting in the fragmentation of a once-continuous forest (Wampach 1988). The high permeability of the agricultural matrix observed in this study suggests that *P. leucopus* has the potential to spread fairly quickly. The high number of ticks found in site D also increases the chances of the Lyme disease vector spreading west along with the mice at a rapid rate. Approximately 20 km northwest of our study area is the St. Lawrence Seaway, which runs along southern Québec connecting Ontario's Great Lakes to the Atlantic Ocean. We can hypothesize that the St. Lawrence Seaway could be an effective barrier and impede the northern expansion of the white-footed mouse. Several high-traffic highways located just before the seaway may likely slow dispersal further (highways 15, 20, and 30). Southeastern Québec has no main water bodies intersecting it in the north-south direction, and the forests are much less fragmented than on the western side of the region (Fig. 1). Although main water bodies are absent, this increased forested landscape may still provide effective control of *P. leucopus* populations.

A larger forested area and increased connectivity between patches attracts a larger variety of species that do poorly in small, isolated forest fragments. Larger and more connected fragments thus become suitable habitat for different species, and biodiversity within the patch increases. Vertebrate species richness positively correlates with forest patch size and negatively correlates with white-footed mouse abundance (LoGiudice et al. 2008), creating a dilution effect: infected ticks feed on a variety of species with low reservoir competence which decreases the chances of the reservoir competent *P. leucopus* from becoming infected (Van Buskirk and Ostfeld 1995). Increased biodiversity will also heighten resource and habitat competition as well as number of predators, thus reducing the white-footed mouse's home range and abundance. Finally, encounter reduction may also arise: a constrained home range decreases the chances of a mouse from encountering a tick or from fighting with an infected mouse and acquiring the bacteria through bodily fluids (Brunner and Ostfeld 2008). Southern Québec has the highest biodiversity level within the province, but also the greatest number of species threatened by forest fragmentation (Auzel et al. 2012). Maintaining high biodiversity levels or increasing biodiversity could provide efficient control on *P. leucopus* population abundance and dispersal, thus reducing the rate of spread of Lyme disease in southern Québec.

Although the white-footed mouse plays an important role as a reservoir for the bacterium that causes Lyme disease, this bacterium also requires the black-legged tick (i.e., the disease vector) for transmission and thus cycle completion. A survey of over 50 sites in southern Québec revealed the presence of ticks in vegetation approximately 100 km north of our study area (Bouchard et al. 2011). Tick populations may reach new regions via their vertebrate hosts, but are most likely dispersed over large geographic distances by migratory birds (Ogden et al. 2010). The success of the establishment of these ticks in their new northern environments will then depend on the suitability of their surrounding climatic conditions (Ogden et al. 2005) in addition to the presence of their hosts. Although ticks are carried further north into the province, their current populations cannot establish themselves successfully due to present climatic conditions (Ogden et al. 2006). Among the 11 populations we sampled, three of them (sites C, D, & E) had a high number of ticks, but site D was the only site that hosted mice and ticks that were infected with *Borrelia*. The risk of exposure to Lyme disease is significantly elevated in fragmented forested landscapes due to their ability to host high densities of white-footed mice which, in turn, are highly competent in transmitting *Borrelia* to the population of ticks residing within the same patch (Allan et al. 2003; Halos et al. 2010). Southern Québec has been observing this trend, and today, the northern edge of tick and Lyme disease emergence is currently in the southern part of the province (Ogden et al. 2010). Although Lyme disease has not yet been detected in sites C & E, these two forest patches are likely to host Lyme disease in a near future due to the high numbers of ticks we recorded at these sites.

The confirmed tick and Lyme disease emergence in southern Québec, coupled with the range expansion and population abundance of the efficient Lyme disease reservoir *P. leucopus*, shows how important it is to merge *P. leucopus* population analyses like ours with ongoing Lyme disease research in Québec. Such combined efforts may then be applied toward disease management strategies within Québec and within other Canadian provinces with similar ecological conditions to help understand how the disease may spread.

## Supporting Information

**Figure S1.** Study area with pie charts showing the ratios of assigned genetic clusters of individuals in each population from STRUCTURE.

**Table S1**. *F*_ST_ values (above the diagonal) for the 11 populations studied and the number of significant results for the Fisher exact test (below the diagonal) for the eleven loci. Significant *F*_ST_ values are indicated by asterisks (^ns^*P* > 0.05, ***P* < 0.01, ****P* < 0.001). Probabilities were adjusted with sequential Bonferroni corrections.

**Table S2.** STRUCTURE results showing [A] the probability values for the number of genetic clusters (L[K] with standard deviation [SD] and ΔK) for mice from Montérégie, Québec, using *K* = 1–12, and [B] the proportion (Q) of every population's genome that is part of each *K* subpopulation (*K* = 2).

**Table S3**. Effective number of migrants (*Nm*) for all mountain populations and their closest fragment (source-sink pairs identified by matching symbols). Total *Nm* is summed as total immigration^a^ and total emigration^b^ rates for each population.

**Table S4**. Gene flow symmetry (GFS) indices between source-sink populations. If gene flow moved asymmetrically from site *i* to site *j*, then we would expect private alleles to accumulate faster at site *i* thus creating a GFS index of greater than 1. Asymmetrical gene flow is observed between two mountain populations (site *i*) and their nearest forest fragment populations (site *j*): higher flow from site D to H and from site C to J.

**Table S5**. Results of the assignment test where the 11 populations in the Montérégie region were considered independent units. The success rate of assignment is indicated as %, and *n* represents the population size.

**Appendix S1.** PCR and primer information.
