# Supplementary figures and images for: Genetic structure of the white-footed mouse in the context of the emergence of Lyme disease in southern Québec
Source: Ecol Evol. 2013 Jun 3;3(7):2075–88. doi: 10.1002/ece3.620 (PMC3728948; doi:10.1002/ece3.620)

Figure S1

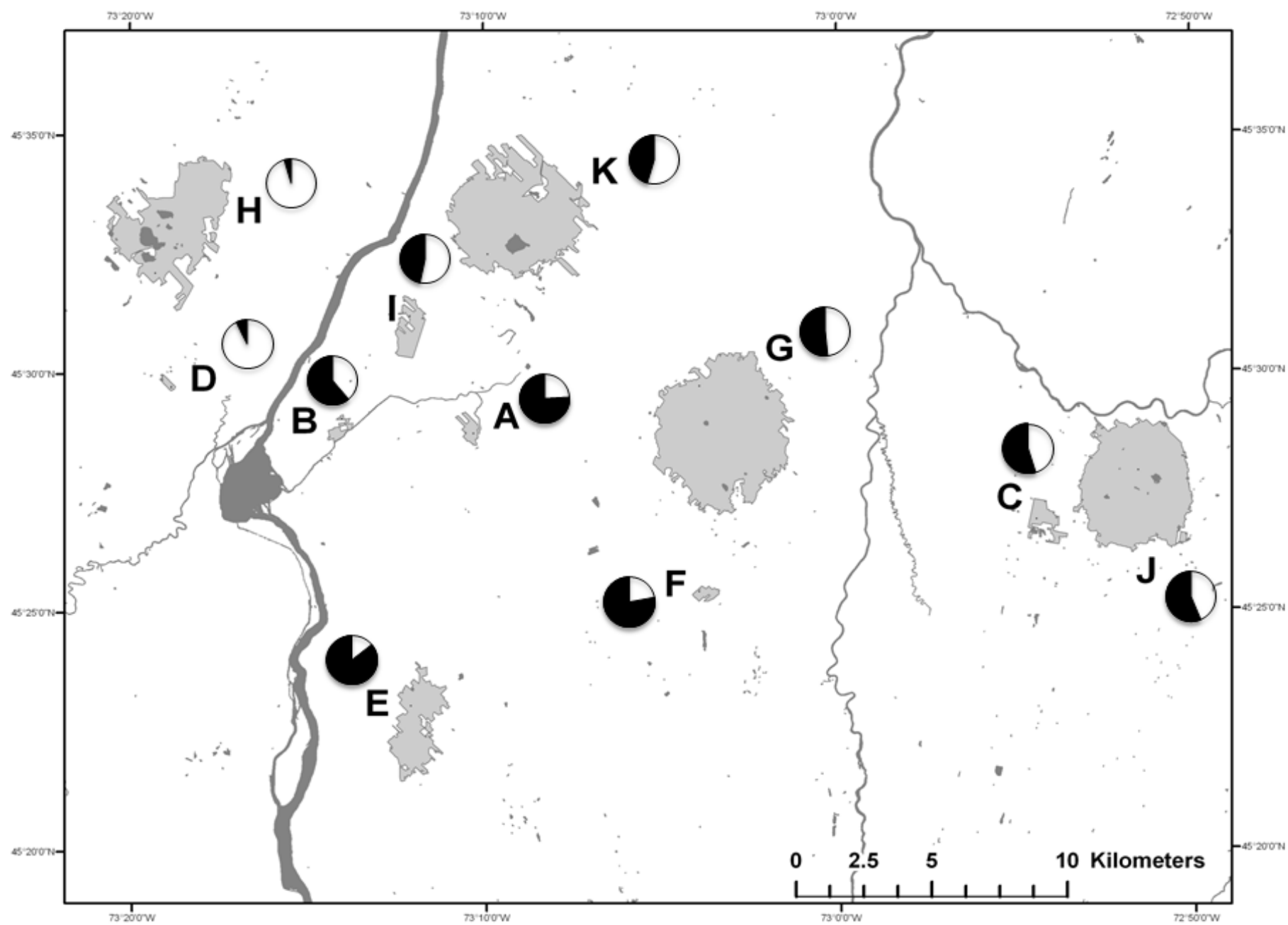

Supplement: Supplementary file 1 [file ece30003-2075-SD1.pdf]
