# Supplementary material for: Genetic structure of the white-footed mouse in the context of the emergence of Lyme disease in southern Québec
Source: Ecol Evol. 2013 Jun 3;3(7):2075–88. doi: 10.1002/ece3.620 (PMC3728948; doi:10.1002/ece3.620)
Supplement: Supplementary file 2 [file ece30003-2075-SD2.docx]

*Table S1*. F_ST_ values (above the diagonal) for the 11 populations studied and the number of significant results for the Fisher exact test (below the diagonal) for the eleven loci. Significant F_ST_ values are indicated by asterisks (^ns^: p>0.05, **: p<0.01, ***: p<0.001). Probabilities were adjusted with sequential Bonferroni corrections.

|  | Site A | Site B | Site C | Site D | Site E | Site F | Site G | Site H | Site I | Site J | Site K |
| --- | --- | --- | --- | --- | --- | --- | --- | --- | --- | --- | --- |
| Site A | - | 0.005  ^ns^ | 0.027*** | 0.054*** | 0.01** | 0.01*** | 0.004  ^ns^ | 0.037*** | 0.008** | 0.021*** | 0.016*** |
| Site B | 0 | - | 0.019*** | 0.039*** | 0.017*** | 0.01** | 0 ^ns^ | 0.026*** | 0.003  ^ns^ | 0.01  ^ns^ | 0.006  ^ns^ |
| Site C | 2/11 | 2/11 | - | 0.037*** | 0.016*** | 0.018*** | 0.011*** | 0.031*** | 0.021*** | 0.009  ^ns^ | 0.01*** |
| Site D | 6/11 | 6/11 | 4/11 | - | 0.048*** | 0.043*** | 0.031*** | 0.021*** | 0.034*** | 0.047*** | 0.038*** |
| Site E | 2/11 | 3/11 | 2/11 | 6/11 | - | 0.006 ^ns^ | 0.01*** | 0.039*** | 0.012*** | 0.01** | 0.015*** |
| Site F | 1/11 | 1/11 | 3/11 | 6/11 | 0 | - | 0.006  ^ns^ | 0.027*** | 0.012*** | 0.01** | 0.012*** |
| Site G | 0 | 0 | 1/11 | 5/11 | 1/11 | 1/11 | - | 0.018*** | 0.002  ^ns^ | 0.003  ^ns^ | 0.0007  ^ns^ |
| Site H | 6/11 | 5/11 | 6/11 | 1/11 | 7/11 | 5/11 | 3/11 | - | 0.023*** | 0.029*** | 0.031*** |
| Site I | 2/11 | 0 | 2/11 | 4/11 | 2/11 | 2/11 | 0 | 6/11 | - | 0.016*** | 0.006  ^ns^ |
| Site J | 0 | 1/11 | 0 | 5/11 | 1/11 | 1/11 | 0 | 4/11 | 0 | - | 0.010  ^ns^ |
| Site K | 3/11 | 1/11 | 1/11 | 6/11 | 3/11 | 2/11 | 0 | 5/11 | 0 | 0 | - |

*Table S2.* STRUCTURE results showing [A] the probability values for the number of genetic clusters (L(K) with standard deviation (SD) and ΔK) for mice from Montérégie, Québec, using K = 1 – 12, and [B] the proportion (Q) of every population’s genome that is part of each K subpopulation (K=2).

| K | L(K) | SD | ΔK |
| --- | --- | --- | --- |
| 1 | -17546.5 | 0.000 | - |
| 2 | -17244.1 | 0.032 | 5401.487 |
| 3 | -17112.5 | 0.106 | 301.789 |
| 4 | -17012.9 | 55.15 | 0.478 |
| 5 | -16939.7 | 14.41 | 5.321 |
| 6 | -16943.1 | 24.32 | 0.464 |
| 7 | -16935.3 | 11.35 | 1.143 |
| 8 | -16940.5 | 24.58 | 0.679 |
| 9 | -16928.9 | 0.117 | 184.957 |
| 10 | -16939.1 | 34.57 | 0.286 |
| 11 | -16939.4 | 24.68 | 0.202 |
| 12 | -16934.7 | 24.24 | 0.194 |

|  | Inferred Clusters | |  |
| --- | --- | --- | --- |
| Population | #1 | #2 | *n* |
| A | 0.26 | 0.74 | 35 |
| B | 0.42 | 0.58 | 32 |
| C | 0.47 | 0.53 | 34 |
| D | 0.91 | 0.09 | 31 |
| E | 0.14 | 0.86 | 34 |
| F | 0.23 | 0.77 | 35 |
| G | 0.49 | 0.51 | 35 |
| H | 0.96 | 0.04 | 37 |
| I | 0.54 | 0.46 | 35 |
| J | 0.44 | 0.56 | 21 |
| K | 0.56 | 0.44 | 38 |

A B

*Table S3*. Effective number of migrants (*Nm*) for all mountain populations and their closest fragment (source-sink pairs identified by matching symbols). Total *Nm* is summed as total immigration^a^ and total emigration^b^ rates for each population.

|  | Site C | Site D | Site F | Site G | Site H | Site I | Site J | Site K | Total Nm^b^ |
| --- | --- | --- | --- | --- | --- | --- | --- | --- | --- |
| Site C |  | 0.31 | 0.35 | 0.30 | 0.26 | 0.38 | 0.26 ^Δ^ | 0.32 | 3.00 |
| Site D | 0.30 |  | 0.18 | 0.28 | 0.28~ | 0.27 | 0.38 | 0.24 | 2.81 |
| Site F | 0.27 | 0.26 |  | 0.31° | 0.27 | 0.26 | 0.43 | 0.25 | 2.96 |
| Site G | 0.36 | 0.31 | 0.38° |  | 0.28 | 0.31 | 0.32 | 0.27 | 3.17 |
| Site H | 0.28 | 0.30~ | 0.28 | 0.25 |  | 0.15 | 0.25 | 0.26 | 2.59 |
| Site I | 0.29 | 0.31 | 0.31 | 0.35 | 0.34 |  | 0.29 | 0.37^#^ | 3.11 |
| Site J | 0.24 ^Δ^ | 0.24 | 0.30 | 0.29 | 0.26 | 0.24 |  | 0.27 | 2.72 |
| Site K | 0.31 | 0.29 | 0.31 | 0.24 | 0.27 | 0.28^#^ | 0.32 |  | 2.82 |
| Total Nm^a^ | 2.87 | 2.90 | 3.14 | 2.91 | 2.81 | 2.67 | 3.24 | 2.75 |  |

*Table S4*. Gene flow symmetry (GFS) indices between source-sink populations. If gene flow moved asymmetrically from site *i* to site *j*, then we would expect private alleles to accumulate faster at site *i* thus creating a GFS index of greater than 1. Asymmetrical gene flow is observed between two mountain populations (site *i*) and their nearest forest fragment populations (site *j*): higher flow from site D to H and from site C to J.

| Site *i* | Site *j* | GFS index | Standard error | 95% confidence interval |
| --- | --- | --- | --- | --- |
| F | G | 1.57 | 0.40 | 0.79 – 2.35 |
| D | H | 1.49 | 0.23 | 1.04 – 1.94 |
| C | J | 2.61 | 0.51 | 1.61 – 3.61 |
| I | K | 1.10 | 0.26 | 0.59 – 1.61 |

*Table S5*. Results of the assignment test where the 11 populations in the Montérégie region were considered independent units. The success rate of assignment is indicated as %, and *n* represents the population size.

| Population | *n* | % |
| --- | --- | --- |
| Site A | 35 | 91.4 |
| Site B | 32 | 59.4 |
| Site C | 34 | 79.4 |
| Site D | 31 | 83.9 |
| Site E | 34 | 70.6 |
| Site F | 35 | 85.7 |
| Site G | 35 | 91.4 |
| Site H | 37 | 97.3 |
| Site I | 35 | 62.9 |
| Site J | 21 | 71.4 |
| Site K | 28 | 73.7 |

**Appendix S1: PCR and primer information**

PCR conditions for the primers used to amplify the 11 microsatellite loci. Amplifications were carried out in 18μL volumes and included 1× reaction buffer, 2 mmol/L MgCl_2_, 2.5 mmol/L of each dNTP, 0.15μmol/L of each primer, 0.5 U (1 U ≈ 16.67 nkat) of *Taq* DNA polymerase, genomic template DNA (50-250 ng per sample), and 13.83 μL of sterile H_2_O. All primers were marked with fluorescent labeling allowing the alleles to be detected during the genotyping process (Schuelke 2000). Polymerase chain reaction (PCR) conditions were similar for all primers except for certain annealing temperatures that varied as follows: *PMl*01, *PMl*03, *PMl*09 & *PMl*12 = 54°C; *PMl*04, *PMl*06 & *PMl*11 = 58°C; *PMl*05, *PLGT58* & *PLGATA70* = 55°C; and *PLGT66* = 57°C. Thermal cycling was carried out on a GeneAmp® PCR System 9700 (Applied Biosystems) under the following conditions: 95°C for 3 minutes; 35 cycles of 95°C for 30 seconds, the primer’s specific annealing temperature for 45 seconds, then 72°C for 45 seconds; and a final extension time at 72°C for 10 minutes.

**Primer References:**

Schuelke M (2000) An economic method for the fluorescent labeling of PCR fragments: A poor man’s approach to genotyping for research and high-throughput diagnostics. *Nature Biotechnology*, **18**, 233-234.
